# Association of Socio-Clinical Factors With Psychological Distress in Parents or Caregivers of Children With Mental Illness

**DOI:** 10.1177/00207640261438843

**Published:** 2026-04-30

**Authors:** Zeruiah Bensingh, Zukiswa Tsolekile De Wet, Marinda Joubert

**Affiliations:** 1 University of Pretoria, Gauteng, South Africa

**Keywords:** caregivers, mental health, depression, anxiety, perceived stress, socioclinical factors, tertiary level psychiatric hospital, South Africa

## Abstract

The COVID-19 pandemic has had a strong psychological impact on family units. This study investigated the contribution of sociodemographic and clinical factors to psychological distress in parents or caregivers of children with a mental illness at a specialist tertiary hospital in Gauteng, South Africa. A structured sociodemographic and clinical questionnaire measuring psychological distress was self-administered to 150 participants. The Perceived Stress Scale (PSS) was used to measure the severity of stress, the Patient Health Questionnaire-9 (PHQ-9) was used to measure the severity of depression, and the Generalized Anxiety Disorder-7 (GAD-7) was used to measure the severity of anxiety. Significant predictors of mental health status include loss of home due to COVID-19 (odds ratio [*OR*: 46.61], 95% confidence interval [CI: 5.81, 1,089.92]), history of psychiatric treatment (*OR*: 11.98, [3.16, 57.22]), perceived decline in quality of life (*OR*: 11.88, [1.20, 185.28]), female sex (*OR*: 3.83, [1.00, 17.7]), and difficulty spending time with extended family (*OR*: 3.09, [1.10, 9.29]). The mental health status of caregivers of children with mental illness during the COVID-19 pandemic was likely influenced by the loss of a home due to COVID-19, a history of psychiatric treatment, a perceived decline in quality of life, the female gender, and difficulty spending time with extended family. Our study demonstrated the intricate interplay of socioclinical factors with depression, anxiety, and perceived stress.

## Introduction

Parents and caregivers of children with a mental illness face substantial psychological burdens, with elevated risks of stress, anxiety, and depression. Caregiver distress is influenced by socio-clinical factors such as gender, psychiatric history, and the availability of social support. Socio-clinical factors have been previously operationalized as housing, income, and family stress (Holman et al., 2020; Thoits, 2011). High levels of parental stress have been associated with depression, reduced emotional involvement, family conflict, and negative child outcomes (Abidin, 1990; Coldwell et al., 2008; McPherson et al., 2009). These dynamics underscore the importance of understanding how sociodemographic and clinical factors shape caregiver well-being.

The COVID-19 pandemic added further strain by disrupting routines, limiting access to support, and increasing financial insecurity. Families with children requiring mental health care were particularly vulnerable, as caregiver burden often involves balancing competing responsibilities and sacrificing personal well-being to meet the needs of the child (Granek et al., 2014; Martinez-Marcos & De la Cuesta-Benjumea, 2015). While pandemic-related stressors such as isolation, stigma, and uncertainty intensified distress (Brooks et al., 2020; Hawryluck et al., 2004), they largely acted as amplifiers of pre-existing vulnerabilities rather than stand-alone causes.

In South Africa, little is known about the psychological well-being of parents and caregivers of children with mental illness, despite evidence that disruptions to routines and support systems negatively affect both caregivers and children (Olive et al., 2021). Addressing this gap is critical, as identifying predictors of caregiver distress can inform targeted interventions for vulnerable families in resource-constrained settings.

This study therefore investigates the association between depression, anxiety, and perceived stress with sociodemographic and clinical factors among parents and caregivers of children with mental illness at a tertiary psychiatric hospital in Gauteng, South Africa.

## Research Methods and Design

### Study Design

This quantitative, cross-sectional analytical study involved a questionnaire survey that was completed during an interview with the parents or caregivers of mentally ill children who attended the child and adolescent clinic at Weskoppies Hospital.

### Setting

The study occurred in the outpatient child and adolescent clinic at a tertiary-level psychiatric hospital in Pretoria West, Gauteng, South Africa. Data was collected.

### Participants

The study population included parents or caregivers (older than 18 years) of children (younger than 18 years) who were mentally ill and who attended the child and adolescent clinic at Weskoppies, a psychiatric hospital in Gauteng, South Africa. In this study, “caregiver” was defined according to the South African Children’s Act (2005) as any person other than a biological or legal parent who has assumed primary responsibility for the care of a child. Parents or caregivers were included if they could read and speak English, could independently complete self-report questionnaires, and had children with a mental illness. Parents or caregivers were excluded if they could not read or speak English. Data was collected between March 2023 and April 2023.

Permission to administer questionnaires targeting the parents or caregivers at the child and adolescent unit was granted by the Chief Executive Officer of Weskoppies Psychiatric Hospital before data collection. Personal consent was obtained. Each participant’s questionnaire was allocated a study number to anonymize the data. The Faculty of Health Sciences Research Ethics Committee, University of Pretoria, approved the study (Reference number: 34/2023). The study was also submitted to The National Health Research Database of South Africa for approval (Reference number: *GP202303 009*).

### Measures

Three self-administered tools were used to screen for, diagnose, and measure the severity of stress, anxiety, and depression. Additionally, a self-administered sociodemographic questionnaire was completed, which recorded information including designation, gender, education level, and marital status (Supplemental Material).

Stress was assessed using the Perceived Stress Scale-10 (PSS-10; Cohen et al., 1983). The PSS-10 is a widely used tool designed to evaluate how various life situations influence individuals’ feelings and perceived stress. It assesses thoughts and emotions experienced over the preceding month. Each of the ten items measures the frequency of stress-related feelings, with total scores ranging from 0 to 40. Scores <13 indicate low stress, 14 to 26 indicate moderate stress, and >27 indicate high perceived stress.

The PSS-10 has demonstrated good internal consistency, with Cronbach’s α values ranging from .74 to .91 in international studies (Cohen et al., 1983) and α = .81 in South African samples (Makhubela, 2020). The scale has been validated and used among adults and students in the Gauteng, KwaZulu-Natal, Eastern Cape, and Western Cape provinces (Hamad et al., 2008; Makhubela, 2020), confirming its cultural relevance and psychometric robustness.

Depression was assessed using the Patient Health Questionnaire-9 (PHQ-9; Kroenke et al., 2001; Spitzer et al., 1999). The PHQ-9 corresponds to the nine diagnostic criteria for major depressive disorder (MDD**)** as defined in the DSM-IV and evaluates depressive symptoms over the preceding 2 weeks. Scores range from 0 to 27, categorized as follows: <5 = minimal, 5 to 9 = mild, 10 to 14 = moderate, 15 to 19 = moderately severe, and ⩾20 = severe depression.

The PHQ-9 has consistently shown high reliability, with Cronbach’s α values ranging from .86 to .89 in international validation studies (Kroenke et al., 2001) and α = .86 among South African primary care patients (Bhana et al., 2015). The instrument has been used effectively in KwaZulu-Natal and Gauteng as a depression screening tool (Bhana et al., 2015; Cholera et al., 2014).

Anxiety was measured using the Generalized Anxiety Disorder-7 (GAD-7) scale (Spitzer et al., 2006). The GAD-7 assesses the presence and severity of generalized anxiety symptoms over the past 2 weeks, based on DSM-IV criteria. Total scores range from 0 to 21, categorized as follows: 0 to 4 = minimal, 5 to 9 = mild, 10 to 14 = moderate, and 15 to 21 = severe anxiety.

The GAD-7 has demonstrated excellent internal reliability, with Cronbach’s α values between .89 and .92 internationally (Spitzer et al., 2006) and α = .89 in South African populations (Kigozi, 2021). The tool has been validated and used in the Free State and KwaZulu-Natal provinces (Naidoo et al., 2020; Truong et al., 2021), supporting its applicability in local contexts.

Collectively, these psychometric instruments have demonstrated robust internal consistency, validity, and cultural adaptability in South African populations. Their brevity, ease of self-administration, and strong psychometric evidence make them suitable for use in diverse, resource-limited healthcare environments.

### Data Analysis

The statistical analysis was conducted using R software (R Core Team, 2020, version 3.6.3). The categorical variables are described as counts and frequencies. The associations between categorical variables were assessed using chi-square tests. If the distribution of the cross-tabulations contained an expected value of less than five, Fisher’s exact test was used. For significant differences, a row-wise paired *z* test was used for post hoc analysis following the omnibus tests (chi-square test or Fisher’s exact test). We checked for multicollinearity using a generalized variance inflation factor approach and used Cook’s distance to remove influential observations. Backward stepwise elimination was applied based on the Akaike information criterion (AIC) to identify the most important explanatory variables. The regression results were tabulated. Statistical significance was set at 5% (*p* < .5).

## Results

Table 1 presents the demographics of the parents or caregivers of the children with a mental illness. The results revealed that most respondents were parents (82.7%), female (76.7%), held a tertiary level of education (50%) and were married or living together (52.7%). The majority of participants in this study were women (76.7%), which likely reflects sociocultural caregiving norms in South Africa, where mothers and female relatives are more often primary caregivers of children with chronic illnesses, including mental health conditions.

**Table 1.** Demographic Characteristics of Parents or Caregivers of Children With a Mental Illness (*N* = 150).

	Overall (*N* = 150) (%)
Designation	
Parent	124 (82.7)
Caregiver	26 (17.3)
Gender	
Male	34 (23.3)
Female	112 (76.7)
Education	
Primary	6 (4.0)
Secondary	68 (45.9)
Tertiary	74 (50.0)
Marital status	
Single	45 (30.4)
Married/living together	78 (52.7)
Widowed	3 (2.0)
Separated/Divorced	22 (14.9)

*Note*. Valid % shown; Mode1 = smallest mode; Nmodes = # of modes.

Table 2 shows the comparisons between Parents (*N* = 124) and Caregivers (*N* = 26) across various measures related to psychological distress and perceived stress. In terms of problem difficulty measured on PHQ9, the majority of participants in both groups rated their difficulties as “Not difficult at all” or “Somewhat difficult.” Parents reported slightly higher proportions of “Very difficult” (20.6%) and “Extremely difficult” (4.7%) compared to caregivers (17.4% and 0%, respectively). Similarly, both groups primarily reported “Not difficult at all” or “Somewhat difficult” on GAD7. “Very difficult” and “Extremely difficult” were reported more by parents (25.8% and 7.5%) than caregivers (9.5% and 0%).

**Table 2.** Comparison of Psychological Distress Among Parents and Caregivers of Children With Mental Illness.

Designation	Parent (*N* = 124) (%)	Caregiver (*N* = 26) (%)	*p*-Value	Overall (*N* = 150) (%)
PHQ9 problem difficulty			Fisher’s, *p* = .475	
Not difficult at all	33 (30.8)	11 (47.8)		44 (33.8)
Somewhat difficult	47 (43.9)	8 (34.8)		55 (42.3)
Very difficult	22 (20.6)	4 (17.4)		26 (20.0)
Extremely difficult	5 (4.7)	0 (0.0)		5 (3.8)
GAD7 problem difficulty			Fisher’s, *p* = .198	
Not difficult at all	30 (32.3)	9 (42.9)		39 (34.2)
Somewhat difficult	32 (34.4)	10 (47.6)		42 (36.8)
Very difficult	24 (25.8)	2 (9.5)		26 (22.8)
Extremely difficult	7 (7.5)	0 (0.0)		7 (6.1)
PHQ9 severity			Fisher’s, *p* = .831	
Severe	14 (11.3)	1 (3.8)		15 (10.0)
Moderately severe	22 (17.7)	4 (15.4)		26 (17.3)
Moderate	20 (16.1)	4 (15.4)		24 (16.0)
Mild	27 (21.8)	6 (23.1)		33 (22.0)
Minimal	30 (24.2)	7 (26.9)		37 (24.7)
Not at all	11 (8.9)	4 (15.4)		15 (10.0)
GAD7 anxiety severity			Chisq., *p* = .189	
Severe	27 (21.8)	3 (11.5)		30 (20.0)
Moderate	24 (19.4)	2 (7.7)		26 (17.3)
Mild	24 (19.4)	8 (30.8)		32 (21.3)
Minimal	31 (25.0)	6 (23.1)		37 (24.7)
Not at all	18 (14.5)	7 (26.9)		25 (16.7)
Perceived stress			Fisher’s, *p* = .320	
High	23 (18.5)	3 (11.5)		26 (17.3)
Moderate	83 (66.9)	16 (61.5)		99 (66.0)
Low	13 (10.5)	6 (23.1)		19 (12.7)
Never	5 (4.0)	1 (3.8)		6 (4.0)

Regarding severity of depression symptoms on PHQ9, the majority of participants fell within “Mild,” “Minimal,” or “Moderately severe” categories. Severe depression was more common in parents (11.3%) than caregivers (3.8%). In terms of severity of anxiety symptoms on GAD7, severe anxiety was reported by 21.8% of parents compared to 11.5% of caregivers. Caregivers were more likely to report “Mild” or “Minimal” anxiety levels. In terms of level of stress perceived by participants, most participants reported “Moderate” stress (66.9% of parents, 61.5% of caregivers). Caregivers were slightly more likely to report “Low” stress levels (23.1% vs. 10.5%).

Overall, the results suggest no statistically significant differences between parents and caregivers in the assessed measures of problem difficulty, severity of depressive and anxiety symptoms, or perceived stress. However, some trends indicate that parents might experience slightly higher levels of severe depression and anxiety compared to caregivers. Caregivers were more likely to report “Minimal” or “Low” levels of difficulty and stress.

Table 3 provides correlation coefficients (*r*) to describe the relationships between PHQ9 scores (depression), GAD7 anxiety scores, and Perceived Stress scores. A very strong positive correlation exists between PHQ9 (depression) and GAD7 (anxiety) scores (*r* = .856; *p* < .001). This means individuals with higher depression levels tend to have higher anxiety levels. A moderate positive correlation is observed between PHQ9 (depression) and Perceived Stress scores (*r* = .552; *p* < .001). This suggests that as depression symptoms increase, perceived stress also tends to rise. Similarly, a moderate positive correlation exists between GAD7 (anxiety) and Perceived Stress scores (*r* = .546; *p* < .001). This indicates that higher anxiety levels are associated with greater perceived stress.

**Table 3.** Association Between Depression, Anxiety, and Perceived Stress.

Variable 1	Variable 2	Correlation	*p*-Value
PHQ9 score	GAD7 anxiety score	.856	<.001
PHQ9 score	Perceived stress score	.552	<.001
GAD7 anxiety score	Perceived stress score	.546	<.001

### PHQ-9 Depression Severity

**Figure 1.**
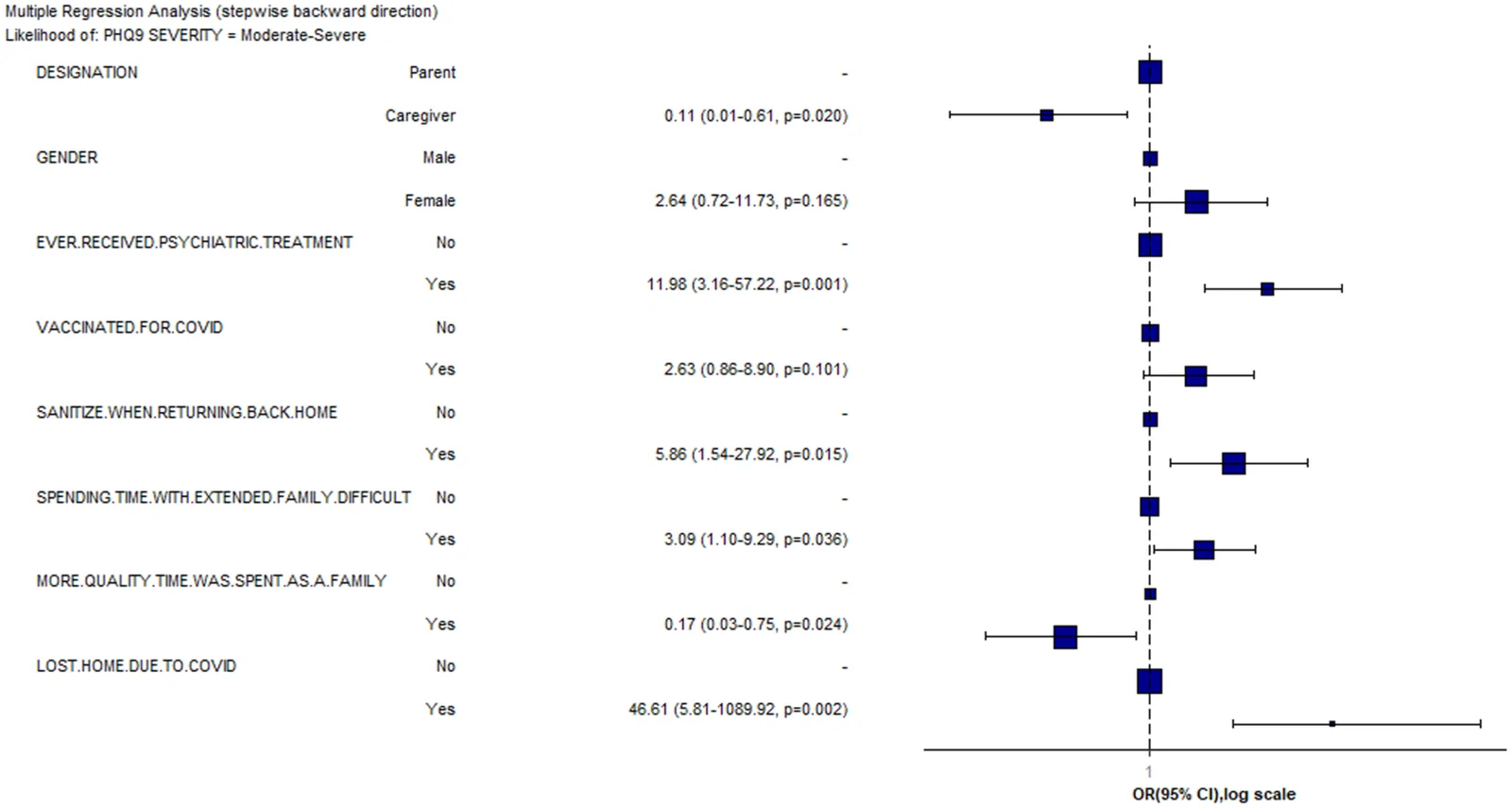
Likelihood of PHQ9 severity.

Figure 1 presents a multiple regression analysis using the stepwise backward method to determine factors associated with the likelihood of PHQ9 Severity = Moderate to Severe (dependent variable). The results are expressed as Odds Ratios (*OR*) with their 95% Confidence Intervals (CI) and *p*-values.

#### Designation (Parent vs. Caregiver)

Caregivers are significantly less likely to report moderate-to-severe PHQ9 scores compared to parents (*OR* = 0.11; 95% CI: [0.01, 0.61], *p* = .020. This suggests that being a caregiver may have a protective effect against higher depression severity.

#### Gender (Male vs. Female)

Females are more likely to report moderate-to-severe PHQ9 scores compared to males, though this association is not statistically significant (*OR* = 2.64; 95% CI: [0.72, 11.73], *p* = .165).

#### Ever Received Psychiatric Treatment (No vs. Yes)

Individuals who have received psychiatric treatment are significantly more likely to report moderate-to-severe PHQ9 scores, indicating a strong association between prior psychiatric treatment and higher depression severity (*OR* = 11.98; 95% CI: [3.16, 57.22], *p* = .001).

#### Vaccinated for COVID (No vs. Yes)

Unvaccinated individuals show a higher likelihood of reporting moderate-to-severe PHQ9 scores compared to vaccinated individuals, but this relationship is not statistically significant (*OR* = 2.63; 95% CI: [0.86, 8.90], *p* = .101).

#### Sanitizing When Returning Back Home (No vs. Yes)

Those who did not sanitize after returning home are significantly more likely to report moderate-to-severe PHQ9 scores, suggesting a potential link between precautionary behaviors and lower depression severity (*OR* = 5.86; 95% CI: [1.54, 27.92], *p* = .015).

#### Spending Time With Extended Family Difficult (No vs. Yes)

Difficulty spending time with extended family is significantly associated with a higher likelihood of moderate-to-severe depression (*OR* = 3.09; 95% CI: [1.10, 9.29], *p* = .036), highlighting the importance of social interactions in mental health.

#### More Quality Time Spent as a Family (No vs. Yes)

Spending more quality time as a family is significantly associated with a lower likelihood of moderate-to-severe depression (*OR* = 0.17; 95% CI: [0.03, 0.75], *p* = .024), indicating a protective factor.

#### Lost Home Due to COVID (No vs. Yes)

Individuals who lost their homes due to COVID are dramatically more likely to report moderate-to-severe PHQ9 scores (*OR* = 46.61; 95% CI: [5.81, 1,089.92], *p* = .002). This indicates a severe impact of such financial and emotional hardship on mental health.

### GAD-7 Anxiety Severity

**Figure 2.**
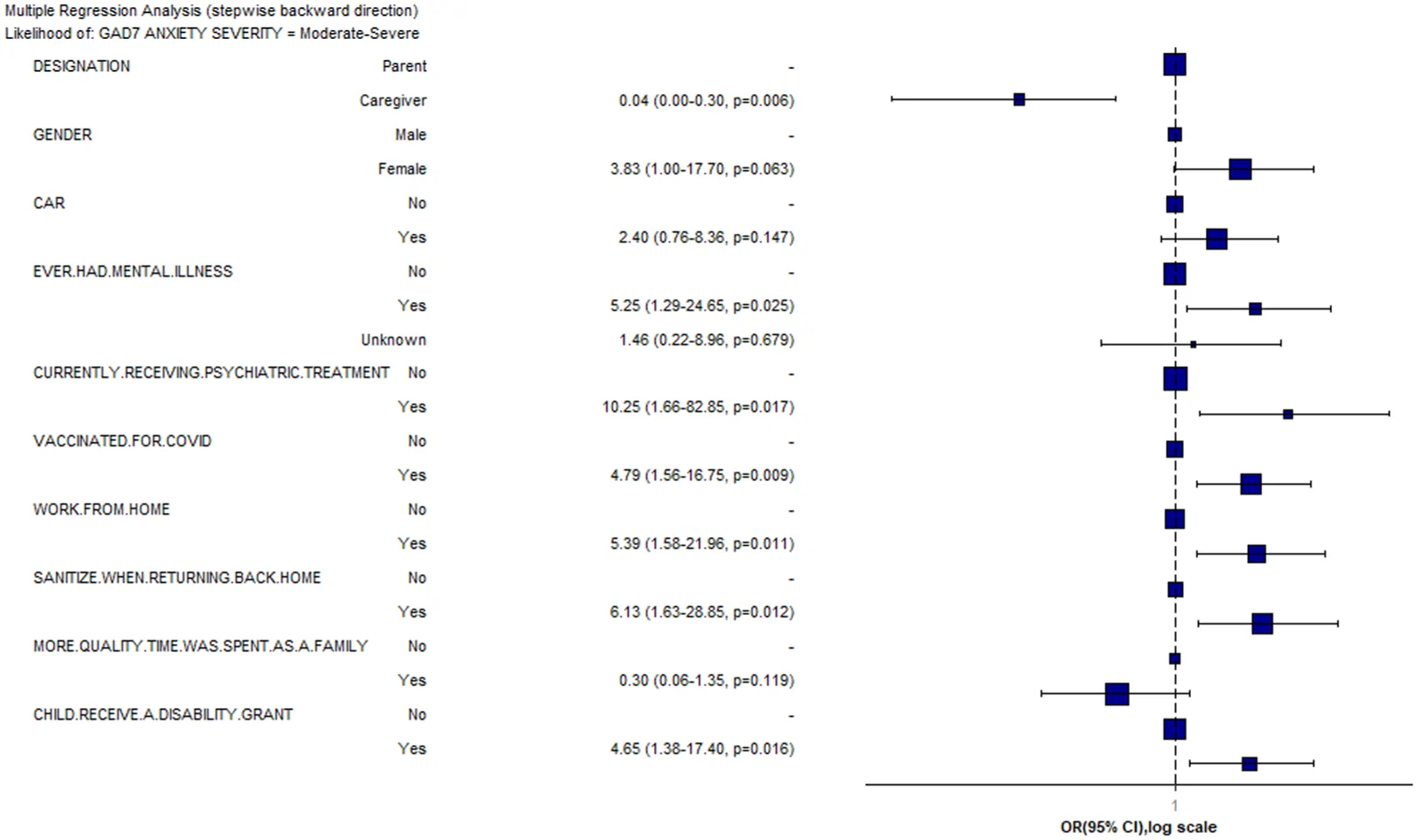
The likelihood of GAD7 anxiety.

Figure 2 presents a multiple regression analysis using the stepwise backward method to assess factors associated with the likelihood of GAD7 Anxiety Severity = Moderate to Severe (dependent variable). The results are presented as Odds Ratios (*OR*) with 95% Confidence Intervals (CI) and *p*-values.

#### Designation (Parent vs. Caregiver)

Caregivers are significantly less likely to experience moderate-to-severe anxiety compared to parents (*OR* = 0.04; 95% CI: [0.00, 0.30], *p* = .006), suggesting that caregiving may serve as a protective factor.

#### Gender (Male vs. Female)

Females are more likely to experience moderate-to-severe anxiety compared to males *OR* = 3.83; 95% CI: [1.00, 17.70], *p* = .063). However, this relationship is not statistically significant (*p* > .05).

#### Car Ownership (No vs. Yes)

Individuals without a car are more likely to experience moderate-to-severe anxiety, but this association is not statistically significant (*OR* = 2.40; 95% CI: [0.76, 8.36], *p* = .147).

#### Ever Had Mental Illness (No vs. Yes)

Individuals with a history of mental illness are significantly more likely to experience moderate-to-severe anxiety (*OR* = 5.25; 95% CI: [1.29, 24.65], *p* = .025), indicating a strong association between past mental health conditions and anxiety severity.

#### Currently Receiving Psychiatric Treatment (No vs. Yes)

Those currently receiving psychiatric treatment are significantly more likely to report moderate-to-severe anxiety (*OR* = 10.25; 95% CI: [1.66, 82.85], *p* = .017), highlighting ongoing mental health challenges in this group.

#### Vaccinated for COVID (No vs. Yes)

Unvaccinated individuals are significantly more likely to experience moderate-to-severe anxiety compared to vaccinated individuals (*OR* = 4.79; 95% CI: [1.56, 16.75], *p* = .009).

#### Work From Home (No vs. Yes)

Individuals not working from home are significantly more likely to experience moderate-to-severe anxiety (*OR* = 5.39; 95% CI: [1.58, 21.96], *p* = .011), suggesting that working from home might offer mental health benefits.

#### Sanitizing When Returning Back Home (No vs. Yes)

Those who did not sanitize after returning home are significantly more likely to experience moderate-to-severe anxiety (*OR* = 6.13; 95% CI: [1.63, 28.85], *p* = .012), indicating a potential link between precautionary behaviors and anxiety levels.

#### More Quality Time Spent as a Family (No vs. Yes)

Spending more quality time as a family appears to reduce the likelihood of moderate-to-severe anxiety (*OR* = 0.30; 95% CI: [0.06, 1.35], *p* = .119), although this relationship is not statistically significant.

#### Child Receiving a Disability Grant (No vs. Yes)

Having a child who receives a disability grant is significantly associated with a higher likelihood of moderate-to-severe anxiety (*OR* = 4.65; 95% CI: [1.38, 17.40], *p* = .016), reflecting the potential stress of caregiving responsibilities in this context.

### PSS-10 Perceived Stress

**Figure 3.**
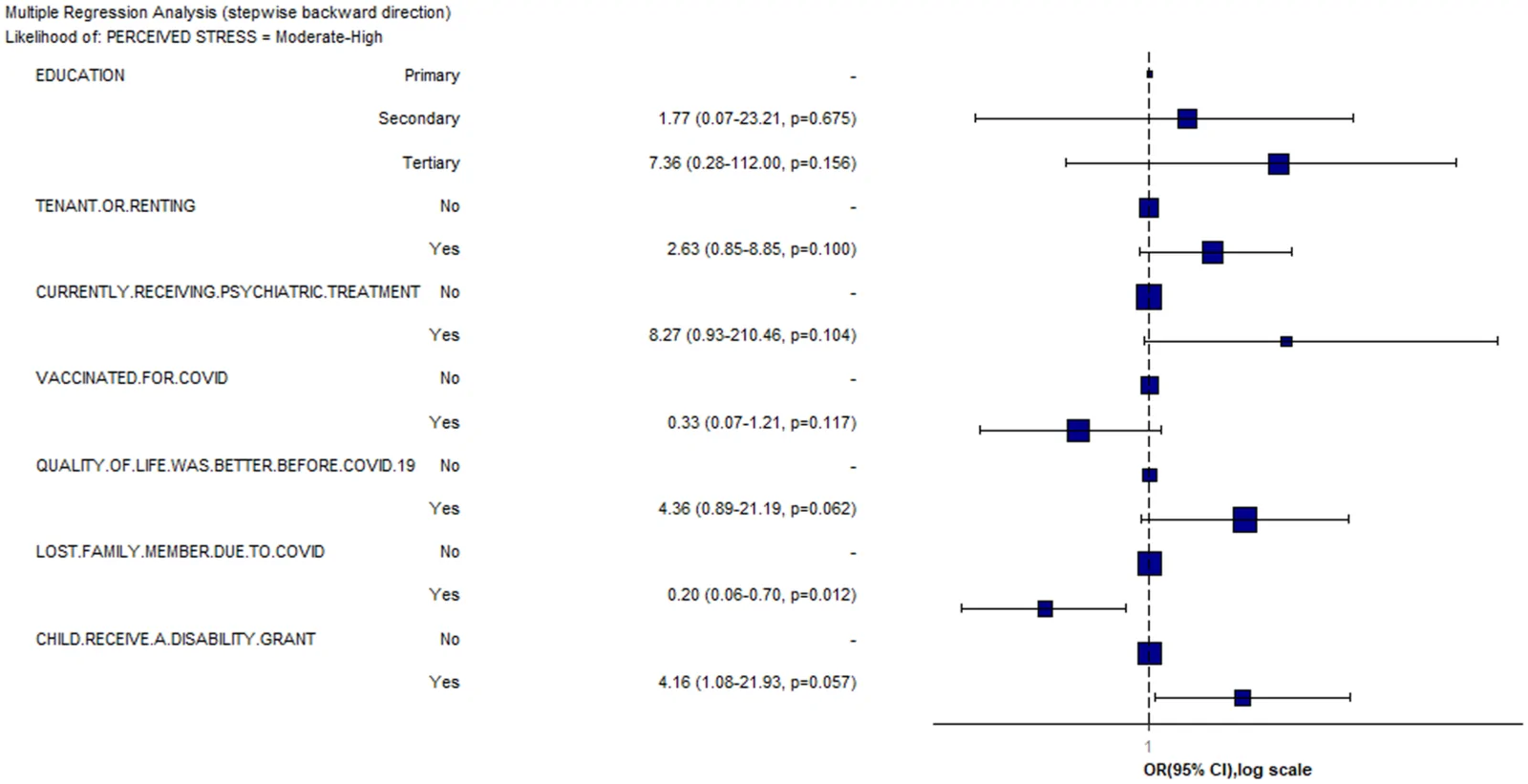
The likelihood of perceived stress.

Figure 3 shows the results of a multiple regression analysis (stepwise backward direction) evaluating factors associated with the likelihood of Perceived Stress = Moderate-High. The results are summarized as follows:

#### Education (Primary as Reference Group)

Education level does not significantly affect perceived stress levels, though tertiary education (*OR* = 7.36; 95% CI: [0.28, 112.00], *p* = .156) shows a higher likelihood of moderate-to-high stress compared to primary education.

#### Tenant or Renting (No vs. Yes)

Renting or being a tenant shows a trend toward higher stress levels, but this association is not statistically significant (*OR* = 2.63; 95% CI: [0.85, 8.85], *p* = .100).

#### Currently Receiving Psychiatric Treatment (No vs. Yes)

Individuals currently receiving psychiatric treatment have an increased likelihood of moderate-to-high perceived stress, but this association does not reach statistical significance (*OR* = 8.27; 95% CI: [0.93, 210.46], *p* = .104).

#### Vaccinated for COVID (No vs. Yes)

Vaccinated individuals are less likely to report moderate-to-high stress, though the relationship is not statistically significant (*OR* = 0.33; 95% CI: [0.07, 1.21], *p* = .117).

#### Quality of Life Was Better Before COVID (No vs. Yes)

Those who perceive their quality of life as better before COVID show a trend toward higher perceived stress, approaching statistical significance (*OR* = 4.36; 95% CI: [0.89, 21.19], *p* = .062).

#### Lost a Family Member Due to COVID (No vs. Yes)

Surprisingly, individuals who lost a family member to COVID are significantly less likely to experience moderate-to-high perceived stress (*OR* = 0.20; 95% CI: [0.06, 0.70], *p* = .012). This could reflect a unique coping mechanism or unmeasured variables related to their circumstances.

#### Child Receives a Disability Grant (No vs. Yes)

Having a child who receives a disability grant is associated with an increased likelihood of moderate- to-high perceived stress, approaching statistical significance (*OR* = 4.16; 95% CI: [1.08, 21.93], *p* = .057).

## Discussion

The findings presented in Tables 2 and 3, along with Figures 1 to 3, provide critical insights into the psychological well-being of parents and caregivers during the COVID-19 pandemic, with a focus on depression, anxiety, and perceived stress levels. The comparative analysis revealed notable differences between these two groups. While most parents and caregivers rated their difficulties on the PHQ9 and GAD7 scales as “Not difficult at all” or “Somewhat difficult,” parents were more likely to report higher difficulty levels, including “Very difficult” and “Extremely difficult.” This trend extended to the severity of depressive and anxiety symptoms, with severe depression and anxiety being more prevalent among parents. This aligns with evidence that the dual pressures of parenting and caregiving during crises exacerbate psychological strain (Patrick et al., 2020; Prime et al., 2020). In contrast, caregivers may benefit from more focused roles and established coping strategies, consistent with literature highlighting resilience among caregiving populations (Schulz & Sherwood, 2008).

### Correlations Between Depression, Anxiety, and Stress

Moderate to strong positive correlations were observed between depression, anxiety, and stress scores. This reinforces the cumulative nature of psychological distress, where symptoms of one condition amplify the others. These findings are consistent with prior research documenting the bidirectional relationship between depression, anxiety, and stress during crises such as the COVID-19 pandemic (Czeisler, 2020; Vindegaard & Benros, 2020).

### Factors Influencing Depression, Anxiety, and Stress

Regression analyses identified several predictors of psychological distress. Caregivers were significantly less likely than parents to report moderate-to-severe depression and anxiety, suggesting a potential protective effect of their caregiving role. Females trended toward higher distress, reflecting gender disparities reported in other studies (Kuehner, 2017).

Pre-existing psychiatric illness and ongoing treatment were strongly associated with elevated depression, anxiety, and stress, underscoring the heightened vulnerability of individuals with prior mental health conditions (Hao et al., 2020; Vindegaard & Benros, 2020). These findings emphasize the importance of targeted interventions and accessible services during crises, in line with World Health Organization recommendations (Jawad et al., 2021).

Behavioral and social factors also shaped outcomes. Sanitizing upon returning home and spending quality family time were protective against distress, likely by fostering a sense of control and support. Conversely, financial hardship, such as housing loss, dramatically increased depression risk, reflecting the profound impact of socioeconomic disruption. Our results are consistent with findings by Gillespie-Smith et al. (2023), who reported heightened distress among UK caregivers of children with neurodevelopmental disabilities during pandemic restrictions. Interestingly, participants who lost family members to COVID-19 reported lower stress levels, which may reflect cultural coping mechanisms or community and spiritual support (Wallace et al., 2020).

### Quality of Life and Stress

Participants reporting a decline in quality of life since the pandemic showed a trend toward higher stress, consistent with evidence that disruptions to stability are major contributors to psychological strain (Holman et al., 2020). Caregivers of children receiving disability grants were also more likely to experience stress, echoing studies documenting the compounded burdens faced by caregivers of children with disabilities during crises (Willner et al., 2020). Financial support may provide some relief, but additional interventions such as respite care, caregiver support groups, and accessible mental health resources are needed.

### Key New Findings From This Study

While the strong correlations between depression, anxiety, and perceived stress observed in our study are consistent with existing literature, several novel findings emerged. First, contextual factors such as loss of housing due to COVID-19 and reduced opportunities to spend time with extended family showed particularly strong associations with psychological distress, highlighting the combined impact of socioeconomic instability and disrupted social support systems. Second, protective factors including working from home, family bonding, and precautionary health behaviors emerged as significant buffers against distress. These findings suggest that interventions should not only target individual mental health symptoms but also address structural and social determinants.

### Implications and Recommendations

These findings underscore the urgent need for interventions that directly address parental stress and its psychological consequences. Parents who juggle dual caregiving and occupational responsibilities should be prioritized for support through accessible counseling services and structured parenting workshops. Gender-sensitive strategies and workplace flexibility are also essential to reduce inequities disproportionately affecting women (Kuehner, 2017). In addition, socioeconomic stressors, particularly housing insecurity, should be mitigated through coordinated financial and housing support initiatives that integrate psychosocial care.

Several action-oriented strategies can be drawn from these results. First, expanding financial and housing assistance for families of children with mental illness would help buffer the destabilizing impact of economic hardship. Second, structured caregiver support groups should be established to reduce isolation, enhance coping skills, and build peer solidarity. Third, family-centered interventions that strengthen bonding and distribute caregiving responsibilities equitably could reinforce resilience at the household level. Finally, flexible workplace policies for parents of children with special health needs would allow families to balance employment with caregiving more sustainably.

Together, these approaches can reduce caregiver burden, strengthen family resilience, and improve the sustainability of care for vulnerable households. Public health strategies should promote protective factors such as family bonding, precautionary health behaviors, and vaccination, which have been shown to reduce psychological distress (Murphy et al., 2021). Expanding telehealth and counseling services remains a priority, particularly for individuals with pre-existing psychiatric conditions (Hao et al., 2020; Jawad et al., 2021). By identifying both risk and protective factors, this study underscores the importance of supporting caregiver well-being to safeguard child outcomes during and beyond crises.

### Limitations, Bias, and Confounders

Several limitations must be acknowledged. The cross-sectional design prevents causal inference, and reliance on self-reported data introduces recall and social desirability bias. Although models adjusted for sociodemographic and clinical variables, residual confounding cannot be excluded, particularly in relation to socioeconomic status and unmeasured aspects of social support. Selection bias may have occurred due to the English literacy requirement and single-site sampling, limiting generalizability. The absence of a control group of caregivers of children without mental illness, as well as the lack of baseline or pre-pandemic measures, further constrains interpretation.

## Conclusion

This study provides valuable insights into the psychological well-being of parents and caregivers during the COVID-19 pandemic, focusing on depression, anxiety, and stress. A comparative analysis revealed that parents experienced higher levels of severe depression and anxiety than caregivers, likely due to the multifaceted stressors of parenting, including managing children’s education and household responsibilities. In contrast, caregivers exhibited greater resilience, possibly due to a more structured caregiving role with fewer competing demands.

Strong correlations between depression, anxiety, and stress underscored the interconnected nature of psychological distress, highlighting the cumulative impact of these challenges during crises. Predictors of heightened psychological distress included pre-existing mental health conditions, ongoing psychiatric treatment, and financial insecurity, such as housing instability. On the other hand, protective factors, including family bonding, precautionary health behaviors, and vaccination, demonstrated a mitigating effect on psychological distress. Interestingly, individuals who lost a family member to COVID-19 reported lower stress levels, potentially indicating post-traumatic growth or unmeasured supportive mechanisms. Gender differences were also notable, with females exhibiting a trend toward higher levels of depression and anxiety, emphasizing the need for gender-sensitive interventions. Additionally, caregivers of children with disabilities faced heightened stress despite financial support, highlighting the need for comprehensive care programs.

Future research should explore the long-term psychological impacts of the pandemic across diverse populations, investigate the role of social and community support, and examine unexpected findings, such as reduced stress among bereaved individuals. Interventions should prioritize mental health support for parents and caregivers, expand telehealth and counseling services, and address gender disparities through flexible workplace policies. Finally, addressing socioeconomic stressors through financial relief and support systems remains critical for mitigating mental health challenges during crises. By addressing these issues, future efforts can build resilience and improve psychological outcomes for vulnerable population groups.

## Supplemental Material

sj-docx-1-isp-10.1177_00207640261438843 – Supplemental material for Association of Socio-Clinical Factors With Psychological Distress in Parents or Caregivers of Children With Mental Illness Supplemental material, sj-docx-1-isp-10.1177_00207640261438843 for Association of Socio-Clinical Factors With Psychological Distress in Parents or Caregivers of Children With Mental Illness by Zeruiah Bensingh, Zukiswa Tsolekile De Wet and Marinda Joubert in International Journal of Social Psychiatry
