# Supplementary material for: Association of Socio-Clinical Factors With Psychological Distress in Parents or Caregivers of Children With Mental Illness
Source: Int J Soc Psychiatry. 2026 Apr 30;72(6):1786–95. doi: 10.1177/00207640261438843 (PMC13569914; doi:10.1177/00207640261438843)
Supplement: Supplementary file 1 [file sj-docx-1-isp-10.1177_00207640261438843.docx]

# Appendices

Appendix 1: Participant Information Document and Consent form

Appendix 2: Data collection Sheet/ Sociodemographic Questionnaire

Appendix 3: Perceived Health Questionnaire

Appendix 4: General Anxiety Disorder Questionnaire

Appendix 5: Perceived Stress Scale

Appendix 1:

PARTICIPANT’S INFORMATION & INFORMED CONSENT DOCUMENT (Attached on separate document)

**Appendix 2:**

**Sociodemographic Questionnaire**

**BI: BASIC INFORMATION**

| **BI-01. Date** |  |  | **BI-02. Subject No** |  | **BI-03. Subject Code** |
| --- | --- | --- | --- | --- | --- |
| DD / MM / YYYY |  |  |  |  |  |

**BI-04. Area**

| **BI-05**  **Designation** | **Parent [1]** |
| --- | --- |
|  | **Caregiver [2]** |

**DM: DEMOGRAPHICS** *(Please tick [√] or circle the appropriate box)*

| **DM-01** | Primary Language Spoken? | | South African English [**1]** | Afrikaans [**2]** | Xhosa [3**]** | Northern Sotho[4**]** | Siswati [5**]** | Tsonga [6**]** |
| --- | --- | --- | --- | --- | --- | --- | --- | --- |
|  |  |  | Sepedi [**7**] | Venda [**8**] | Tswana [9] | Sotho[10] | Zulu[11**]** |  |

| **DM-02** | **Gender** *(Do not read)* | Male **[1]** | Female **[2]** |
| --- | --- | --- | --- |

| **DM-04** | Highest level of Education you attended? | No schooling [**0]** | Primary [**1]** | Secondary [**2]** | Tertiary [**3]** |
| --- | --- | --- | --- | --- | --- |

| **DM-05** | What is your **marital status**? | Single[**1]** | Married/living together [**2]** | | Widowed [**3]** |
| --- | --- | --- | --- | --- | --- |
|  |  |  |  |  | Separated/Divorced [**4**] |

**HE: HOUSEHOLD SOCIO-ECONOMIC STATUS (***Please tick [√] or circle the appropriate box)*

| **HE-01** | How many **people** currently live (**EAT AND SLEEP**) in this **household**, including you? |  |
| --- | --- | --- |

|  | **Does your household have :** | Yes | No |
| --- | --- | --- | --- |
| **HE-02a** | **Home-ownership?** | 1 | 2 |
| **HE-02b** | **Tenant/Renting** | 1 | 2 |
| **HE-02c** | **Electricity?** | 1 | 2 |
| **HE-02d** | **Cell phone?** | 1 | 2 |
| **HE-02e** | **Car?** | 1 | 2 |

| **HE-03** | Occupational Status | | | Employed [**1]** | Unemployed [2] | Part Time Jobs [3**]** |
| --- | --- | --- | --- | --- | --- | --- |
|  |  |  |  | Retired [**4**] | Receiving Social/Disability Grant [**5**] | |

|  | **Household sources of income:** |  |
| --- | --- | --- |
| **HE-04a** | **Salary** |  |
| **HE-04b** | **Grants** |  |
| **HE-04c** | **Income from a business** |  |
| **HE-04d** | **Remittances (Payment as a bill, invoice or gift)** |  |
| **HE-04e** | **Pension** |  |
| **HE-04f** | **Part Time Jobs** |  |

| **HE-05 Total combined Household income for 1 month** |  |
| --- | --- |
| **HE-05a** | **Below R2500 [1]** |
| **HE-05b** | **More than R2500 [2]** |

**PH: PSYCHIATRIC HISTORY (***Please tick [√] or circle the appropriate box)*

| **PH-01** | Have you had a mental illness ever? | Yes **[1]** | No **[2]** | **Unkown [3]** |
| --- | --- | --- | --- | --- |

| **PH-02** | Have you ever received psychiatric treatment? | Yes **[1]** | No **[2]** |
| --- | --- | --- | --- |

| **PH-03** | Are you currently receiving psychiatric treatment? | Yes **[1]** | No **[2]** |
| --- | --- | --- | --- |

| **PH-04** | Diagnosis |  | Unkown **[1]** |
| --- | --- | --- | --- |

| **PE-05** | Treatment Regimen | | | | Antipsychotics[**1]** | Mood Stabilizers [2] |
| --- | --- | --- | --- | --- | --- | --- |
|  |  |  | Antidepressants3] | Stimulants[4] | Sedatives [5] | Other [6] |

| **PE-06** | **Name of treatment** |  |
| --- | --- | --- |

**FH: FAMILY HISTORY(***Please tick [√] or circle the appropriate box) -Children*

**PH: PSYCHIATRIC HISTORY (*Please tick [√] or circle the appropriate box)*H: PSY**

| **FH-01** | Number of Children |  |
| --- | --- | --- |

| **FH-02** | **Gender of Child** | Male **[1]** | Female **[2]** |
| --- | --- | --- | --- |

| **FH-03** | Age of child receiving Mental Health Treatment? | Early Childhood  (2-5 years) [**1]** | Middle Childhood  (6-11 years) [**2]** | Early Adolescence  (12-18 years) [**3]** |
| --- | --- | --- | --- | --- |

| **FH-04** | Diagnosis |  | Unkown **[1]** |
| --- | --- | --- | --- |

| **FH-05** | Are they attending a School | Yes **[1]** | No **[2]** |
| --- | --- | --- | --- |

| **FH-06** | If Not, who looks after them in the day? |  |
| --- | --- | --- |

| **FH-07** | Treatment Regimen | | | | | Antipsychotics[**1]** | | Mood Stabilizers [2] |
| --- | --- | --- | --- | --- | --- | --- | --- | --- |
|  |  |  | Antidepressants3] | | Stimulants[4] | Sedatives [5] | | Other [6] |
| **FH-08** | **Name of treatment** | | |  | | |  |  |

**C HISTORY (***Please tick [√] or circle te box)*

**CF: COVID FACTORS(***Please tick [√] or circle the appropriate box)*

| **CF-01** | Have you had COVID? | Yes **[1]** | No **[2]** |
| --- | --- | --- | --- |

| **CF-02** | How many people in your immediate family have had COVID? | One **[1]** | **>One[2]** | Unkown **[3]** |
| --- | --- | --- | --- | --- |

| **CF-03** | Have you been vaccinated? | Yes **[1]** | No **[2]** |
| --- | --- | --- | --- |

**CQ: COVID LOCKDOWN(***Please tick [√] or circle the appropriate box)*

| **CQ-01** | Since Schools were closed, were you the main caregiver for your children? | Yes **[1]** | No **[2]** |
| --- | --- | --- | --- |

| **CQ-02** | Did you work from home? | Yes **[1]** | No **[2]** |
| --- | --- | --- | --- |

| **CQ-03** | Did you have to take care of elderly parents ? | Yes **[1]** | No **[2]** |
| --- | --- | --- | --- |

| **CQ-04** | Did you sanitize shoes, groceries, and change of clothes when returning back home? | Yes **[1]** | No **[2]** |
| --- | --- | --- | --- |

| **CQ-05** | Did you find not being able to see/ spend time with extended family difficult? | Yes **[1]** | No **[2]** |
| --- | --- | --- | --- |

| **CQ-06** | Did you miss being able to do social activities? | Yes **[1]** | No **[2]** |
| --- | --- | --- | --- |

| **CQ-07** | Did you still receive your full salary? | Yes **[1]** | No **[2]** |
| --- | --- | --- | --- |

| **CQ-08** | Did you panic buy groceries | Yes **[1]** | No **[2]** |
| --- | --- | --- | --- |

| **CQ-09** | Did you exercise? | Yes **[1]** | No **[2]** |
| --- | --- | --- | --- |

| **CQ-10** | Did you feel your quality of life was better before COVID-19? | Yes **[1]** | No **[2]** |
| --- | --- | --- | --- |

| **CQ-11** | 9) Did you feel that more quality time was spent as a family? | Yes **[1]** | No **[2]** |
| --- | --- | --- | --- |

| **CQ-12** | Did you feel your quality of life was better before | Yes **[1]** | No **[2]** |
| --- | --- | --- | --- |

| **CQ-13** | What were some of the coping mechanisms you used to keep your child/children occupied during quarantine? | | | | TV [**1]** | Exercise [2] |
| --- | --- | --- | --- | --- | --- | --- |
|  |  |  | Internet [3] | Reading [4] | Playing Games [5] | Other [6] |

**LO: LOSSES(***Please tick [√] or circle the appropriate box)*

| **LO-01** | Have you lost any family members due to COVID? | Yes **[1]** | No **[2]** |
| --- | --- | --- | --- |

| **LO-02** | Have you lost your job due to COVID? | Yes **[1]** | No **[2]** |
| --- | --- | --- | --- |

| **LO-03** | Have you lost home due to COVID? | Yes **[1]** | No **[2]** |
| --- | --- | --- | --- |

**GR: GRANT(***Please tick [√] or circle the appropriate box)*

| **GR-01** | Does your child receive a disability grant? | Yes **[1]** | No **[2]** |
| --- | --- | --- | --- |

| **GR-02** | Do you receive a grant for caregivers? | Yes **[1]** | No **[2]** |
| --- | --- | --- | --- |

**Appendix 3:**

**SUBJECT NUMBER:____________**

**Appendix 4:**

**SUBJECT NUMBER:____________**

**Scoring GAD-7 Anxiety Severity**

**Appendix 5:**

**SUBJECT NUMBER:____________**

**Scoring PSS :**
